# Towards new realities: implications of personalized online layers in our daily lives

**DOI:** 10.1515/icom-2024-0017

**Published:** 2024-06-18

**Authors:** Eelco Herder, Laura Stojko, Jannis Strecker, Thomas Neumayr, Enes Yigitbas, Mirjam Augstein

**Affiliations:** Department of Information and Computing Sciences, 8125 Utrecht University, Utrecht, The Netherlands; Department of Computer Science, University of the Bundeswehr Munich, Neubiberg, Germany; Institute of Computer Science, University of St. Gallen, St. Gallen, Switzerland; Research Group Personalized Environments and Collaborative Systems, 394086 University of Applied Sciences Upper Austria, Hagenberg, Austria; Faculty of Electrical Engineering Computer Science and Mathematics, Paderborn University, Paderborn, Germany

**Keywords:** personalization, recommendation, adaptation, hci, realities

## Abstract

We are currently in a period of upheaval, as many new technologies are emerging that open up new possibilities to shape our everyday lives. Particularly, within the field of Personalized Human-Computer Interaction we observe high potential, but also challenges. In this article, we explore how an increasing amount of online services and tools not only further facilitates our lives, but also shapes our lives and how we perceive our environments. For this purpose, we adopt the metaphor of personalized ‘online layers’ and show how these layers are and will be interwoven with the lives that we live in the ‘human layer’ of the real world.

## 1 Introduction

In “Sapiens”, Yuval Noah Harari^1^ argues that human society is an “incredibly complex network of stories” that forms a layer on top of the ‘real’ world of nature. Where wild animals directly live in forests or grasslands and are directly exposed to cold, heat, or rain, humans live in houses, cities, and countries. Moreover, the ‘human’ layer defines to a large extent how we behave or are expected to behave: for instance, Harari argues that money, religion, laws, and brands are just stories that we tell each other. However, these stories work, because we all believe in them and act accordingly.

Similarly, computer systems and particularly the (mobile) Web arguably have created online layers that are placed upon our society, the human layer. Many people have a profile in one or more social networks, most companies provide online services and contact with governmental institutions largely takes place online. On the street, people navigate using their smartphones, while perhaps being engaged in a discussion via a communication app. These activities in the (personalized) ‘online layer’ have been shown to impact our abilities in wayfinding^2^ and our perception of the ‘human reality’. The increasing acceptance and influence of mixed reality interfaces will most likely make the influence of this online layer in our daily lives even larger.

**Figure 1:**
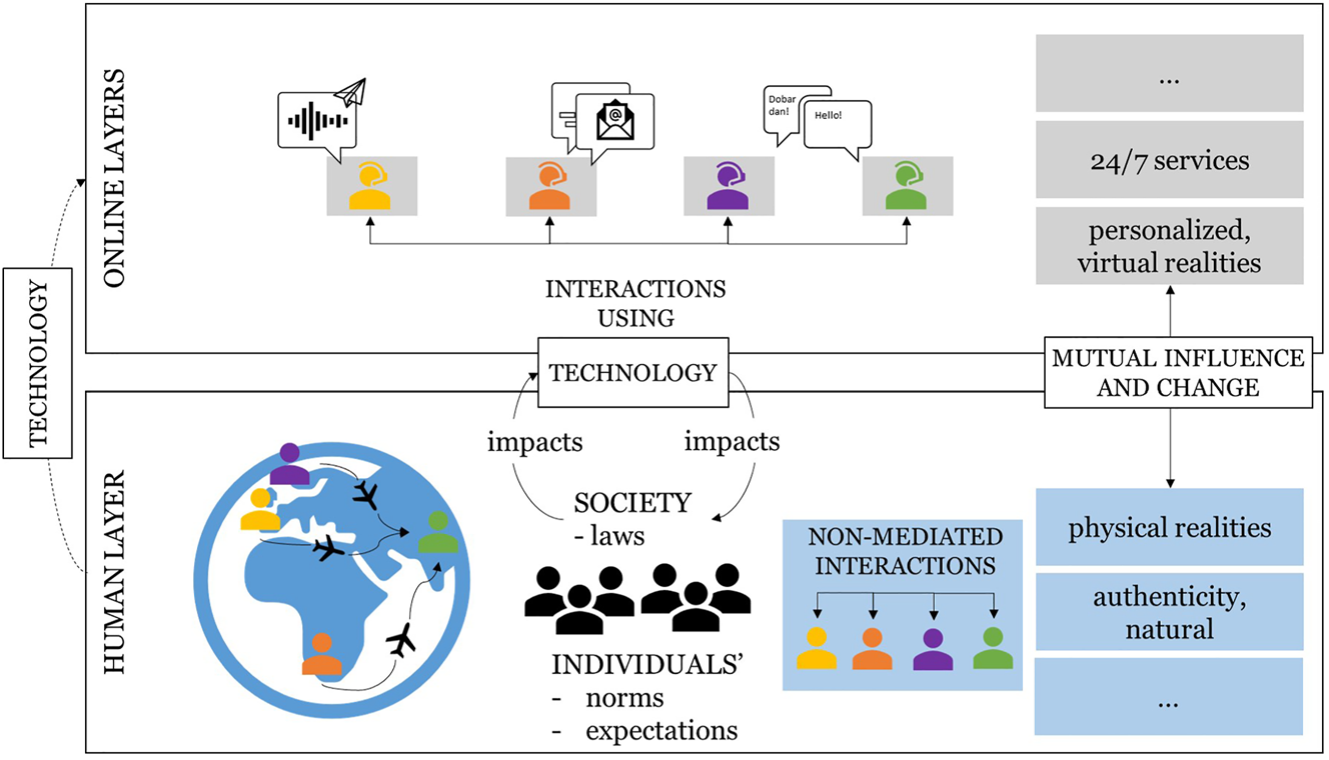
Depiction of connected layers: technology has an impact on society and vice versa and adds a part to the human layer that connects us to the online layers. Each layer has specific characteristics and impacts interaction styles. While the human layer is about non-mediated interactions in a shared physical reality (blue), the online layer enables personalized, virtual realities specific for each person (grey).

The visualization in Figure 1 shows an example of the connection between the human and online layer, their characteristics as well as an example of how social interactions can change. As we will further elaborate in this article, these two layers constitute two human-created dual realities in which we largely spend our lives, on top of the ‘real’ world of nature. We will also discuss how these two layers impact one another.

In this article, we investigate the current state of personalization from this perspective and extrapolate current trends and thoughts. By doing so, we aim to provide several possible scenarios on how the future will develop in this respect and how far we will go. How will the ‘online layers’ develop, connect to our ‘human’ layer, and impact our daily lives? What are the potentials to strive for and challenges that we can expect in the near or more distant future? Our focus will be specifically on the potential effects of technology on individuals’ daily lives, choices, and behavior; for an in-depth discussion on socio-ethical aspects and privacy issues, we refer to works such as Refs. 3–5.

This article is structured as follows. Initially, we discuss the ‘new’ realities that can be created by personalized online layers, followed by a discussion of current challenges, opportunities, and expectations of these. That is succeeded by ideas on how we can use this development to our advantage and a detailed discussion of future scenarios concerning technologies that can assist or take over parts of communication and collaboration. Drivers for successful future development in this regard are identified and we conclude with a potential outlook and considerations for future personalization and adaption in our favor.

## 2 “New” realities created by personalized online layers

In his novel “The Every”, Dave Eggers projects a dystopian future in which our daily lives are heavily influenced and controlled by supposedly useful and benevolent apps, created by a Big Tech monopolist.^6^ At this point in time, many of those apps may appear too far-fetched and unethical to many of us, such as Friendy, which assigns a numerical value to the quality of friendship and trustworthiness of one’s friends by analyzing facial expressions, eye contact, and vocal intonations. However, we believe that many HCI researchers can imagine how, for instance, society can become ready to adopt and accept an app that provides instant feedback on how your conversation partner feels about you and what you say.

As stated above in the introduction, in this article, we use the metaphor of personalized, online layers to investigate their impact on society. In this section, we will discuss the long-term impact of technological developments in the past years or decades that created personalized ‘online layers’ that we live in, just as we do in Harari’s ‘human layer’.

It should be noted that it is not just technology that has an impact on society, but that societal developments have an impact on the development of technology as well (Figure 1); these feedback loops create long-term effects that can only be foreseen to a limited extent.^7^ As a result, societal norms and expectations are likely to shift as well, with gradual steps leading to perhaps radically different conceptions, laws and norms.^8^

As a first well-known example, consider how the telephone has shaped our society and the way we communicate. Before the invention of the telephone, most communication between friends and family took place whenever they would meet. In the past century, phone calls have become increasingly more common, which enabled and supported long-distance relationships, but also partially replaced physical meetings. In recent years, social media and messenger apps enabled us to constantly keep in touch and provide updates. As a result of this information exchange in the *online layer*, physical meetings – such as birthday parties – have transformed from opportunities to catch up with everyone (as a necessity) to a moment where one can simply continue the already ongoing conversation (or play a board game instead).^9^ ^–^ ^11^

A more recent example is the online workplace, which has penetrated our lives in the past decades, particularly during the COVID-19 pandemic. Since the Industrial Revolution, work has become associated with a physical office or factory that one had to commute to. With the spread of networking apps and video conference systems, particularly white-collar workers experience work as an activity that largely takes place online, with the opportunity to visit the physical office if desired or needed. Abstracting from practical benefits and drawbacks, ‘work’ has transformed into something that may always be present in the personalized online layer and that needs to be actively ignored if one wants to separate work from private life.^12^ ^,^ ^13^

As a final example, consider the real and long-term impact that online shopping has on our living environment. Right now, most physical stores, particularly chain stores, have online stores as well. Buying something has been transformed from visiting a store to opening a website or an app. This can be done anytime from anywhere. In a sense, shopping used to be a social activity, but is now a private online activity, with a multitude of online stores providing us a private, personalized shopping experience. Many people consider this convenient, but it adds to our time spent in the online layer instead of the ‘urban layer’. Many city centres already experience the result of this change and started rethinking their function from a shopping area to a social experience.^14^

What all examples have in common is that all involved people interact simultaneously with one another in the human layer, while relying on personalized support in their personalized online layers, which provides guidance that is largely based on their individual profiles and previous interactions with various applications,^15^ as illustrated in Figure 1. In the next sections, we will explore opportunities and challenges that arise with the ever-increasing user reliance on the online layer.

## 3 Current challenges, opportunities, and expectations for the online layer

With the increasing advancements in spatial computing as well as mixed reality (MR) software and technology, the online layer is getting more and more realistic and immersive. This means that the online layer provides similar ways of natural interactions and communications as in the ‘human layer’, which leads to a higher feeling of presence compared to the classical web and mobile applications, which already often are perceived as immersive.

As the introduction of the Apple Vision Pro, Apple’s first spatial computer has shown, current extended reality (XR) devices can merge the ‘human layer’ with the online layer and bring different realities together. This leads to the fact that the online layer is collecting more and more usage data about each of us using connected technologies. In 2022, we produced 103.66 zetabytes of data and by 2027, this amount will grow to 284.3 zetabytes.^16^

This trend of *datafication* shows that the increasing influence of our personalized online layers and the integration of the different (online and real-world) realities results in producing more digital data about ourselves. Using these resources unlocks new potential, especially in terms of big data, personalization, and many other data-driven disciplines. This vast data pool allows us to learn much more about each of us and identify patterns that enable more precise personalization and thus create a great user experience with technology.^17^ ^,^ ^18^ At the same time, datafication and quantification are likely to make us rethink life in a data-driven manner,^19^ potentially transforming sports, leisure reading, or even social interaction into activities that can (and should) be optimized with ad-hoc personalized support.

Conversely, the ‘human layer’ of our real world plays an increasingly important role in our personalized ‘online layers’ as well. It is to be expected that the user interfaces (UIs) that we interact with can be adapted and personalized in near real-time based on a broad spectrum of sensor input. Personalization traditionally relied on large amounts of data and needed considerable time and prolonged, authentic usage to become reliable, for example, in giving suggestions or in adapting content to users’ needs and preferences. In previous years, improved computing power, advanced cloud services, widespread device interconnectivity (IoT), as well as great strides in sensor and tracker technology could be witnessed.

The abundance of computing devices as foreseen by Weiser already in 1991^20^ and the advent of the ubiquity era^21^ are manifestations of those trends. As is typically suggested in the literature on ubiquitous computing (e.g., Ref. ^22^), contemporary interaction paradigms more often rely on bodily awareness, skills, and the spatial and social context. Humans employ different modalities and senses for interaction which can serve as an input to personalization through sensor detection.^23^

These developments paved the way for interrelating inferential data about human-computer interaction beyond prompting users to input text on a keyboard or tracking their mouse movements. Furthermore, with advanced machine learning approaches in combination with the factors mentioned above, behavioral patterns will be detected ever more reliably leading to more robust input for personalizing the *online layer*. Lastly, Large Language Models (LLM) and generative text-to-image systems allowed first considerations around generative UIs (e.g., Refs. 24–26) and it is conceivable that instead of explicit (written or visual) prompts by users, implicit data might be used as a surrogate for prompts to facilitate the instant and continuous adaptation of (mixed reality) UIs.

We expect a switch towards increased *ad-hoc personalization* within the ‘online layer’, based on implicit data coming from sensors (first approaches concerning biosignals are, e.g., Refs. 27,28), effectively reducing the necessity for explicit data collection and preventing issues connected to cold start. The result will be vastly improved ad-hoc estimation of user needs, user preferences, and user context that might find themselves reflected in steadily adapting UIs that help us navigate, communicate, and accomplish other things in the real world.

Without technical mediation, the physical reality so far serves as an objective ground truth that enables some level of *shared experience* in a common reality,^29^ as our perception and interpretation of reality represent the basis for knowledge exchange and the same understanding of terms and concepts. By contrast, personalization in our online layers will likely lead to differing views, with as a result lack of mutual understanding and users suffering from tunnel vision (the online counterpart has been popularized as ‘filter bubbles’^30^). It should be emphasized that our actions in the online layer are just as real as actions in the physical layer, as each may have direct consequences in the other realm as well: as observed in Section 2, online social networks do affect our friendships and relationships, positively and negatively.

Furthermore, if we consider that the increasing capabilities and pervasiveness of MR devices^31^ facilitate pervasive MR applications that may personalize the perception of any aspect of our physical reality, the common base that the physical reality provides may erode. We may end up perceiving different ‘realities’ – not just in a philosophical sense, but in a very real and perceptual one as well. This would make the interaction with others highly challenging, as it would mean a loss of ‘shared worlds’.^32^

As an example, online navigation systems have already transformed how we navigate in (big) cities.^2^ Even people who have lived in a city for a long time – or who are planning to do so – do not necessarily need to learn about the city’s physical layout (streets, living areas, landmarks) anymore, as they can simply follow their personalized routes projected upon the physical city. Building upon this already existing practice, it is easy to imagine how landmarks and shops of interest will be highlighted and others blurred out, resulting in radically different perceptions of the same city.

The emergence of such personalized perceptions of physical reality would require humans to develop an awareness that multiple realities may exist in which the same object may have different properties (cf. Ref. ^33^), different meanings, and different connotations. If everyone insists that their personalized perception is the only true one without being aware that it may not be the case, interpersonal communication will become a lot harder than it already is.

To mitigate such potentially harmful consequences of immersive and pervasive personalization, applications that personalize any aspect of one’s perception should transparently inform users which parts of their perception are personalized,^34^ ^,^ ^35^ and give them the agency to control these adaptions.^36^ ^,^ ^37^ Also, personalizing an interface based on inter-personal or group-related data (cf. Refs. ^38^ ^,^ ^39^) or the sharing of personalized content across users^40^ ^,^ ^41^ might make such pervasive personalization more societally beneficial.

## 4 Future scenario: communication and collaboration

By means of a scenario for the development of communication and collaboration, we demonstrate how this activity of daily life has developed in the past and will develop in the future, considering the depiction of the human and online layers. Additionally, we take a glimpse at the future potentials and developments of this activity over the next 10 years. Particularly, we will explore the impact of technologies for automated adaptation, translation, and text generation that can be employed *during* conversations, meetings, and other gatherings. We believe that this scenario is exemplary for many situations where humans rely on technological support while acting and interacting in the ‘human layer’.^1^

Already nowadays, a part of the ‘online layer’ is communication and collaboration, which no longer predominantly take place strictly face to face, but increasingly remote and also hybrid. Communication and collaboration on the human layer are often supported, mediated, or even replaced by collaboration and communication tools, including social media and instant messaging apps, and video conferencing. As briefly discussed in Section 2, such tools for (interim) communication may alter the nature and purpose of real-world meetings and gatherings as well.

Successful interaction with people from other countries requires the same language for communication, in many cases this is English.^42^ The majority of the world’s population are not native English speakers and thus they have to learn a new language and translate from their native one.

Using our native language(s) in communication conveys a feeling of security, as it is easier for us to express ourselves. Technological tools such as Babelfish can support this translation process already nowadays, e.g. Ref. ^43^, but to what extent can automated translation services support intercultural communication, and enhance or impair this experience?

There are already studies analyzing the cultural suitability of machine translations.^44^ Showed how effective it can be in considering the cultural context. In intercultural communication, for example, the model of Hall,^45^ differentiates preferences in communication into high-context or low-context context styles. Nowadays, technology can assess the personal communication style and use this information to personalize the translation and even adapt to the preferred communication style, e.g., high- or low-context.

Furthermore, translation technologies could support the preservation of minority languages and dialects as there is no ‘need’ to adapt to a common language. This requires the technology to be broader and more considerable of minority languages and dialects. If native languages and dialects are preserved to a greater extent, the mix of languages around us will increase, and with it the interest in cultural traditions that enable people to feel ‘at home’.^46^ This can lead to a challenge, as intercultural competencies are essential for respecting and understanding other cultures and living all together in peace.

The downside of this trend may be the higher risks of getting ‘lost in translation’ and cultural misunderstandings due to translation quality. It goes without saying that any translation, automatic or not, may lead to slight changes in meaning or connotation. This can be extremely dangerous in vital areas such as health and law.^47^ Consequently, it will become important to be able to judge whether a translation is of sufficient quality. More fundamentally, as demonstrated by recent developments in LLMs, AI-generated text, translations or context may be very convincing, especially, as conversation support for instant definitions, glossaries, clarifications, etc. However, they are not necessarily right or helpful, as they are produced with insufficient understanding of the user context.^48^

It is expected that the ability of AI to be involved in human-like reasoning will continue to improve, but it will most likely remain one of the main weaknesses of (deep learning) AI.^49^ Finally, conversations do not only take place in a particular (physical) context, they also usually serve a *pragmatic* goal. Even though AI is quite good at dealing with dialogues on a physical, empirical, syntactical, and semantic level, humans will most likely continue to greatly outperform AI in terms of pragmatics (subtly communicating intentions or understanding subtly communicated intentions).^50^

Automated support during conversations may not be limited to automatic translations, but may also include smart add-ons, such as automatically retrieved pictures or profiles of people that a person refers to, calendar events, or references to Wikipedia. Similar to already existing practices in e-books, these additional cues have the potential to make conversations more efficient and to avoid misunderstanding. On the other hand, however, such add-ons may inadvertently *introduce* misunderstanding as well. Another scenario is to outsource some parts of communication to chatbots. In sales negotiations, this technology is already being used, either in the form of one-way, such as online sales chatbots, or even two-way, such as automated agents negotiating with one another on the stock market.

Ironically, despite the many possibilities for rich, multi-language conversations and understanding, it may well be that we actually will end up with everyone using simplified language, perhaps even still English to increase translation quality, as a common way of communicating to avoid misunderstandings and loss of meaning in translation.^51^ For better translation results and for avoiding possible ambiguity, people may tend to decrease the complexity of content which can also lead to a loss of quality in the end. One reason may be that AI tools may have rich syntax and semantics, but that lack of understanding of pragmatics and constructivism hinders them from being *really helpful*.

To ensure successful collaboration and communication between the combination of the ‘human layer’ and several personalized ‘online layers’, we must steer the development of technological inventions in our favor. This means not blindly trusting the translation or results of AI agents, but being aware of the risks and potential failure of such technology and what it can and cannot do for us. To be able to evaluate the quality of the translation, especially in highly relevant contexts, such as medicine or law, basic language skills are required for judgment of the results.

Therefore, there is a need to educate people in digital literacy especially AI literacy, such that people are capable of differentiating AI-generated content from physical or ‘real’ objects and people, an endeavor which is getting ever more difficult.1
https://www.theguardian.com/world/2024/feb/05/hong-kong-company-deepfake-video-conference-call-scam, last access February 10th, 2024. Here, also the designers and developers of personalized, AI-enabled applications have a responsibility to provide transparency about which content is personalized or adapted, and to give users the agency to customize their experiences. Additionally, governmental organizations can create legal boundaries by defining policies and regulations such as the proposed AI Act of the European Union^52^ to ensure a responsible, user-friendly, and accessible usage of AI and AI-enabled tools in everyday life.

## 5 Future perspectives: handling ‘online layers’

In the previous sections, we have discussed the implications of ongoing, real-time technological support during our daily activities. We have highlighted several exciting new opportunities that may arise in the near or in the more distant future. We have also discussed several possible downsides that we need to anticipate.

The Internet and particularly the mobile Web are still relatively young technologies. As the scenarios in Section 2 demonstrated, the use and adoption of technology – and therewith its purposes and impact – may considerably change over time. Some of these changes can be easily anticipated, but most changes will be the result of long-term feedback loops between users, society, and technology.^7^ Even though the exact details of these effects may be hard to predict, overall trends can be recognized.

In this article, we have used the metaphor of personalized ‘online layers’ that continuously support us in navigating our daily lives in what Harari called the ‘human layer’.^1^ We have argued that while enabling, facilitating, or simplifying actions, such as driving through a city or communicating with colleagues or clients, they may also fundamentally reshape these actions and our perception of what constitutes a city or social relationships.

Transferring simple tasks and non-life-threatening decisions to technology can support us by relieving us of this responsibility and creating capacity for productive and creative work. We can reduce the time spent on boring tasks that nobody wants to take care of. This potential increases ethical discussions regarding the responsibility for recommendations and the (positive as well as negative) consequences that they may have, if accepted.

Seen from a more theoretical perspective, the online layer allows us to outsource and automate or support a significant part of our day-to-day decision-making. For instance, car navigation support transforms the complexity of a city into a comprehensible map with routes to follow. In most cases, this is helpful and allows us to comfortably engage in our daily activities by simply following instructions. However, this comes with several limitations that we need to be aware of.

One of these limitations concerns the inherent weakness of AI in terms of slow, ‘system-2’ decision-making.^49^ According to Kahneman’s^53^ theory of bounded rationality, people mostly engage in fast ‘system-1’ thinking, with routine responses to situations that we are sufficiently familiar with. By contrast, unexpected situations or emergencies call for conscious, step-wise deliberation (‘system-2 thinking’, in terms of Kahneman). As unpredictable situations happen every day, our online layers should keep us alert and ready to take responsibility ourselves in situations where our support tools fall short.

A further limitation concerns the loss of opportunities if we largely or fully let our lives be led by recommendations and personalized support. For instance, navigating through a city by merely following instructions lowers our cognitive burden, but at the same time, it may make us blind to the interesting landmarks or opportunities for entertainment that we pass by, and therefore prevents us from actually *engaging* in what the city, the ‘human layer’, has to offer. Recommender systems and other personalized support are well equipped for suggesting and supporting things that we already know, but this very activity may hinder us from engaging in something new. Therefore, our online layers should keep us aware of the necessity – and our ability – to explore new opportunities by means of *active decision-making*.^7^

In addition to automatizing and supporting our daily lives, many technologies also keep track of our activities and quantify them in the form of dashboards, logs, and reports – as already discussed in Section 3. Currently, users have various apps for activities as varied as running or reading. For both activities, it has been observed that ‘outsourcing’ and quantifying our goals, typically have a positive impact on our productivity, but a negative impact on our enjoyment of these activities.^54^ For instance, to a certain extent, various apps and websites have already transformed the act of reading, which was mainly an enjoyable pastime, into ‘being productive’, targeting ‘knowledge benefits’ and ‘reading goals’.

We believe that society is facing and will face the need to find a healthy balance between the benefits that technology in our online layers provides in terms of convenience and productivity, and the drawbacks that they may have on the actual enjoyment of our activities that ultimately take place in the human layer – be it communicating, navigating, shopping, exercising or reading.

## 6 Conclusions

In the past decades, our daily lives have become supported by numerous tools, apps, sites, and online services. Constant flows of personalized suggestions, recommendations as well as targeted summaries or automatic translations form what we call our personalized *online layer*. This online layer creates an individual lens on our daily (social) environment, which eventually may fundamentally *reshape* how we live and communicate with one another.

The *human layer* is rich and complex. Our online layers reflect this real-world complexity but in a highly individualized manner. When the two of them need to collaborate (such as in technology-mediated conversations), the only workable shared common denominator may turn out to be very basic and simplistic – not just because of the inherent technological limitations, but also because of the way humans are likely to make use of this support.

Trusting technological decisions for unimportant easy tasks without explanation needed, where we can just ‘believe’ the result for adaptation and recommendation without questioning, will make our lives easier but also bears a risk of giving technology too much power.

It is important to consider the advantages and opportunities that technology in the online layer has to offer us, but it is just as important to be aware of its limitations and the negative effects that it may bring to society as well as to individual users. It is also important to monitor who will profit from this development and which people are at risk of losing out. Therefore, it is more important than ever to raise AI awareness and foster digital literacy, to ensure that we collectively will be empowered to actively steer, try out, and reflect on all (personalized) functionality that can be offered by the ‘online layer’ that accompanies us in our lives in the ‘human layer’.
